# Relationship Satisfaction and Infidelity-Related Behaviors on Social Networks: A Preliminary Online Study of Hispanic Women

**DOI:** 10.3390/ejihpe10010023

**Published:** 2019-12-15

**Authors:** Juan Aníbal González-Rivera, Francisco Aquino-Serrano, Emily M. Pérez-Torres

**Affiliations:** 1 School of Behavioral and Brain Sciences, Ponce Health Sciences University, 388 Zona Industrial Reparada 2, Ponce, PR 00716, USA; emilyperez@psm.edu; 2 San Juan Campus, Carlos Albizu University, 151 Calle Tanca San Juan, PR 00901, USA; faquino675@sunmail.albizu.edu

**Keywords:** infidelity behaviors, social networks, relationship satisfaction, relationship ambivalence, emotional intimacy, sexual satisfaction

## Abstract

The purpose of this online study was to develop an explicative model regarding the origin of infidelity-related behaviors on social networks for Hispanic women. We propose that sexual satisfaction and emotional intimacy have a direct impact on the satisfaction of couple relationships, and an indirect impact in the development of infidelity-related behaviors on social networks. To investigate this proposal, we used a non-probabilistic sample of 341 Hispanic women living in Puerto Rico. Statistical analyses confirmed that satisfaction and ambivalence in couple relationship completely mediate the association between sexual satisfaction and infidelity-related behaviors on social networks, as well as the relationship between emotional intimacy and infidelity-related behaviors on social networks. Overall, women who practice infidelity-related behaviors on social networks showed less sexual satisfaction, less emotional intimacy, less relationship satisfaction, and greater ambivalence. Our results provide theoretical and empirical evidence on how infidelity-related behaviors on social networks develop in couple relationships, and these results could help to inform possible forms of prevention and intervention.

## 1. Introduction

Empirical studies on the impact of social networking on the well-being of monogamous couples in Puerto Rico (and Latin America) have been limited. This might seem surprising considering that Puerto Rico has an Internet penetration rate of 85.2%, one of the highest rates in Latin America and the Caribbean [1]. Some researchers in other countries have perceived wide access to cyberspace, particularly social media, as a condition that facilitates the exercise of behaviors associated with infidelity [2,3,4]. The characteristics of individuals, the interactions between them, and the perceptions each individual has about their relationship and their partner have been studied as risk factors [4,5,6].

It is important to ask, and it is important that we clarify from the outset, the reasons why infidelity through digital media should be studied independently of traditional face to face infidelity. Although there are parallels in variables related to both behaviors, Internet-related vulnerabilities have seven specific aspects that are unique [7]. These are: (1) behaviors that are perceived as inappropriate in society tend to be more acceptable when engaged in over the Internet, (2) the Internet is easily accessible, (3) it is economically affordable, (4) it allows anonymity, (5) it facilitates an approximation of the interactions of everyday life, (6) it provides ambiguity in defining problematic behaviors, and (7) it allows people to accommodate their needs in terms of their actual “I” versus their ideal (or digital) “I.”

Digital media worsens the complexity of formulating consistent and clear criteria for judging the infidelity behaviors that already exist outside digital platforms [8,9,10]. While we know that infidelity behaviors on social media also contain sexual and emotional variants—some of which are easily defined, such as flirting and intimate conversations—others may be more controversial, such as remotely chatting with someone, even with strangers [2,3,4,8,10]. On the other hand, researchers have detailed how certain behaviors that might be considered problematic are not perceived and described as such by certain groups. For example, some people who practice cybersex behind their partners’ backs have been found to rationalize and justify their behaviors under the pretext of the absence of physical contact [5]. For this reason, each researcher has been obliged to develop their own criterion of the behaviors they will subject to scrutiny in quantitative research [11].

In this online study, we examined infidelity-related behaviors on social networks to associate different variables with their occurrence in a sample of Hispanic women. In particular, we assessed the impact of satisfaction on couple relationships, and how it is affected by sexual satisfaction and emotional intimacy. We analyzed these variables while taking into account ambivalence in the couple relationship, an individual quality that nuances how a woman interprets interactions with her partner. Several studies have found that, in the United States and in other countries, it is women who often recognize the negative impact of direct and indirect use of social networks on their interpersonal relationships [12,13,14,15]. That is, it is women who tend to experience and more continuously identify the interfering and possibly destructive effects of social networks on couple relationships. In fact, both the evolutionary perspective [16] and the double-shot hypothesis (sociocultural perspective) [17] argue that men and women differ in the ways they perceive infidelity due to differences in socialization. For this reason, in this first study, we have decided to study the behavior of these variables specifically in women, with a view to developing more robust studies in the future that would include both men and women.

### 1.1. Sexual Satisfaction and its Influence on Couple Relationships

For many people, sexuality is a fundamental part of a relationship. This has proven true for men and women, from young people to those beyond middle age, both married and unmarried [18,19,20]. The impact of this factor is of such a magnitude that it is considered one of the integral elements of stability and satisfaction in a relationship [19,20,21]. There is even evidence that changes in sexual satisfaction predict changes in satisfaction with the relationship [19]. However, sexuality is just one of the factors that contribute to relationship satisfaction, as the complexity of human interactions prevents satisfaction from being minimized only to this aspect. For example, other contributing factors may include equity in the distribution of tasks and in decisions on the household economy [22]. However, considering all of the above, it is difficult to question the close relationship between sexual satisfaction and the appreciation of the relationship.

However, is it sexual satisfaction that produces a pleasurable relationship, or is it the quality of the relationship that motivates a fulfilling sex life? Suffice to say that the nature of this association is questionable [22,23]. Although there are theoretical models to justify both perspectives, there is no reliable empirical evidence to establish the dominance of one over the other [23]. Sexual satisfaction is a complex construct that includes physical and emotional pleasure, as well as a subjective assessment of a person’s sex life [21]. Some studies have concluded that the frequency of sexual activity is positively correlated with sexual satisfaction [20,23], along with the variety of sexual activities [24]. In addition, acts of physical intimacy such as petting, touching, and hugging have been observed to affect sexual satisfaction, especially in long-term married couples [20].

### 1.2. Emotional Intimacy and Relationship Satisfaction

Intimacy has two aspects: sexual and emotional. While sexual intimacy is associated with affection in general, contact, physical closeness, and sexual activity [25], emotional intimacy is tied to a feeling of closeness with another person that leads to a tendency to self-reveal to the other. A high level of emotional intimacy will be reflected by a high degree of trust among members of a relationship, loving behaviors, and validating interactions [26]. Although it is usual to make a connection between intimacy and sexuality in the everyday context, in the clinical environment it has been observed that couples satisfied with their sex life do not always feel a marked emotional bond. Likewise, feelings of attachment and emotional connection do not indicate sexual satisfaction [27]. In this study, we will limit intimacy to its emotional side to distance this variable from those that might be more closely related to sexual satisfaction, as discussed above.

Emotional intimacy is an element that contributes powerfully to satisfaction in the relationship [25,28]. Some authors have even concluded that emotional intimacy could contribute more to satisfaction with the relationship than sexual intimacy by itself [25,29]. Recent studies have found that young heterosexual couples who pursued intimacy in their relationship reported higher satisfaction than those that gave more importance to self-exploration and identity development as part of the process of get to know each other [29]. This could be said for both men and women. While some research points out that there are marked gender differences in the scope of emotional intimacy in the satisfaction of the relationship [19,27], others deny that it is a moderating factor, either directly or indirectly [25,30].

### 1.3. Ambivalence in the Relationship

One element that has been little researched and linked to infidelity-related behaviors is the level of ambivalence in the relationship. Ambivalence in the relationship can be defined as the feeling an individual has, whether positive or negative, about their bond with that other person and how they expect the bond to develop in the future [31]. Ambivalence occurs in intimate relationships when there is a coexistence of opposing emotions and desires towards the other person that creates an uncertainty about being in the relationship. Research has shown that higher levels of relationship ambivalence are associated with greater ambiguity in partners’ roles and responsibilities among cohabiting couples [32,33]. These feelings may be the basis for increased instability in cohabiting relationships [33]. According to McDaniel et al. [4], individuals are more likely to employ behaviors related to infidelity when they feel more ambivalent about their stable partner. This same author conducted a study in the United States which indicated that low satisfaction in the relationship and a high level of ambivalence were significantly related to infidelity behaviors through social networks [19]. That is to say, people who feel ambivalent in their couple relationships are more likely to practice behaviors of emotional infidelity through social networks. This research will provide further evidence on how ambivalence is involved in the practice of behaviors associated with infidelity in social networks.

### 1.4. Relationship Satisfaction and Infidelity-Related Behaviors

Cyber infidelity is defined as the act of having an affective/emotional or physical encounter with another person other than your primary romantic partner through social media. It is important to clarify that the spectrum of infidelity, including cybernetics, link together three different dimensions of behaviors classified as infidelity: explicit, ambiguous, and misleading [34]. Explicit behaviors refer to specific behaviors typically associated with physical or sexual infidelity (e.g., practicing sexting with another person). Ambiguous behavior refers to certain behaviors that are not clearly associated with infidelity, but in which there may be a possibility of betrayal (e.g., not allowing their partner to have access to their electronic devices). Usually, these behaviors serve as a preamble to explicit behaviors. Finally, misleading behaviors are characterized by lying or hiding information from the partner (e.g., lying or hiding with whom they share, talk to, or interact with on social networks). The literature confirms that ambiguous and misleading behaviors are more common and acceptable than explicit behaviors [9], even more so when they occur in a cyber-friendly manner [35]. It is important to clarify that when we talk about infidelity-related behaviors, we are referring particularly to the ambiguous and misleading behaviors associated with infidelity on social networks. The importance of studying such behaviors lies in their potential to be indicators and precursors of explicit behaviors of cyber infidelity [4,34,35].

There is evidence that infidelity can be emotional, sexual, or a combination of both [36]. Emotional infidelity may include a relationship through social media, a relationship with a co-worker, or a long-distance relationship, while sexual infidelity may include sexual intercourse with people at work or other types of sexual activity [37]. These categories are not mutually exclusive, because a sexual connection can be transformed into an emotional connection with the passage of time, especially in the case of women [36].

In terms of relationship satisfaction, infidelity-related behaviors, which may include emotional and sexual infidelity, are highly linked to individuals not being satisfied in their relationship [38,39,40]. Other studies have suggested that attitudes towards infidelity are significantly important, as people who show more liberal stances on the issue of infidelity are more likely to be unfair to their partner [41]. A low level of satisfaction in the relationship could also be related to an individual’s lack of commitment to their relationship. This is because satisfaction is an element that influences the level of commitment that the person has; therefore, as there is a decrease in satisfaction, there is a greater tendency to engage in infidelity behaviors [38].

### 1.5. Purpose of the Study

**Figure 1:**
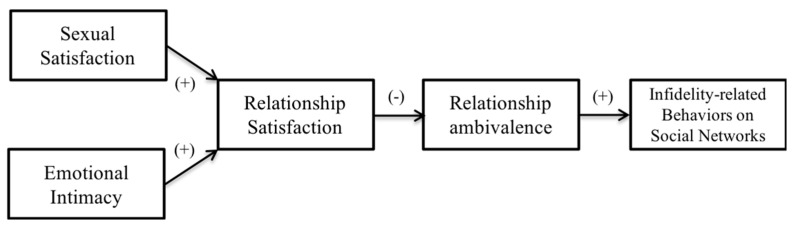
The hypothesized mediation model used for this research.

To date, no study in Latin America has examined infidelity-related behaviors in social networks by associating them with other typical variables of couples’ relationships in order to develop an explanatory model for these behaviors in Hispanic women. To achieve this, the following research objectives were developed: (a) to examine whether low sexual satisfaction and low emotional intimacy have any adverse effect on partner satisfaction and ambivalence, and (b) to examine whether sexual satisfaction and emotional intimacy indirectly promote the exercise of infidelity-related behaviors on social networks. We expect the results to reflect that relationship satisfaction and ambivalence positively and significantly mediate the association between sexual satisfaction, emotional intimacy, and infidelity-related behaviors on social networks (see Figure 1).

## 2. Methods

### 2.1. Research Design

To achieve the objectives of this study, a non-experimental design of a cross-section correlational-causal type [42] was used, examining whether sexual satisfaction and emotional intimacy have any direct effect on partner satisfaction and indirect effect on infidelity-related behaviors on social networks. Data collection was carried out by questionnaires through the PsychData platform, using a paid ad on the main social networks as a method of recruitment, including on Facebook, Instagram, Twitter, Google+, and WhatsApp. This announcement directed participants to the online survey, where they read the informed consent, which reported the following: (a) the purpose of the study, (b) the inclusion criteria, (c) the voluntary nature of the study, (d) the possible risks, and (e) the right to withdraw from the study at any time. To protect the privacy of the participants, the questionnaires were completed anonymously (participants did not have to provide their name, phone number, or email) and participants were able to print a copy of the informed consent if desired. The participants were not financially compensated for their time. All subjects gave their informed consent for inclusion before they participated in the study. The study was conducted in accordance with the Declaration of Helsinki, and the protocol was approved by the Institutional Review Board (IRB) of Carlos Albizu University in San Juan, Puerto Rico (Code: Fall 17-07).

### 2.2. Participants

Originally, 392 Hispanic women agreed to participate in this study and started to answer the questionnaires. However, 51 women left the study before completing 50% of the survey items, consequently they were removed from the sample. The final sample was composed of 341 Hispanic women; of which 323 answered 100% of the survey items. The average age of the participants was 33.95 (SD = 7.53) and the average number of years of cohabitation with their partners was 7.52 (SD = 6.77). In Table 1 we present the sociodemographic data of the sample. To participate in this study, we established the following inclusion criteria: being a resident of Puerto Rico, being 21 years of age or older, and having a heterosexual relationship of cohabitation (married or free union).

### 2.3. Measurement

Sociodemographic Data: To identify the sociodemographic characteristics of the sample, we developed a general data questionnaire composed of relevant data such as age, sex, academic preparation, type of relationship, and annual income.

Relationship Assessment Scale (RAS) [43]: The RAS is a seven-item generic measure of relationship satisfaction. The answers were rated on a 5-point Likert scale, ranging from 1 (poor) to 5 (excellent); items 4 and 7 were reverse scored (e.g., In general, how satisfied are you with your relationship? How often do you wish you had not gotten into this relationship? In general, how satisfied are you with your relationship?). In the present study, the Spanish version of the RAS was adapted for females. Average scores ranged from 1 to 5, while total scores ranged from 7 to 35. The higher the score, the more satisfaction and value was attributed to the relationship and towards their partner by the person (α = 0.92).

Subjective Sexual Satisfaction Scale [44]: This instrument consisted of 20 items organized on a four-point Likert scale ranging from 1 (totally disagree) to 4 (totally agree) (e.g., I consider my sex life to be very exciting; I am satisfied with the amount of sex I practice per week; I feel satisfied with the frequency of my orgasms). The scale consisted of four factors: subjective assessment, emotional aspect, sexual execution, and self-image. The possible range was 20 to 80 points. The higher the score, the higher the person’s sexual satisfaction (α = 0.89).

Infidelity-Related Behaviors on Social Networks Inventory [35]. This inventory evaluates ambiguous and misleading behaviors associated with infidelity on social networks, such as hiding information, keeping secrets, and developing emotional intimacy with others, among others. It consists of eight items organized on a six-point Likert scale ranging from 1 (totally disagree) to 6 (totally agree) (e.g., Sometimes, I prefer to hide things from my partner that I share with other people online or on social media; I have had some conversations by text message or on social networks that I prefer to hide from my partner; I prefer that my partner does not have access to my social networks; It would make me uncomfortable for my partner to read the conversations I have with other people through text messages or on social networks). The lowest score that can be obtained in the final version is 8 and the highest is 48, such that a higher score indicates a greater incidence of behaviors related to cyberinfidelity (α = 0.93).

Emotional Intimacy in Couple Relationships Scale [45]: This instrument consists of 10 items organized on a four-point Likert scale ranging from 1 (totally disagree) to 4 (totally agree) (e.g., Generally, my partner understands my concerns and personal situations; I would like my partner to show me more interest and emotional closeness; I feel confident enough to tell my partner anything that happens to me). The scale consists of two factors: emotional closeness and emotional distance. The lowest score that can be obtained is 10 and the highest is 40, where high scores suggest high emotional intimacy (α = 0.90).

Relationship Ambivalence: To measure this variable, we developed five items that could predict a possible rupture. To create the items, we used the taxonomy of marital instability developed by Booth and Edwards [46]. We performed an exploratory factor analysis using the maximum likelihood extraction method with an oblique rotation. The analyses showed a one-dimensional structure explaining 58% of the variance (see Table 2). The Kaiser-Meyer-Olkin test supported the adequacy of the sample data for the analysis, KMO = 0.761. Bartlett’s test of sphericity was meaningful, χ^2^ (10) = 585.069, *p* < 0.001, demonstrating that correlations between items were significantly different from zero, and this was an additional indicator of adequacy for factor analysis. All of the items obtained discrimination indexes (*r_bis_*) greater than 0.30. The scale obtained an internal consistency index of 0.81 Cronbach’s alpha. All five items were arranged on a five-point Likert scale ranging from 1 (nothing) to 5 (totally) (e.g., I feel ambivalent or unsure about continuing in the relationship with my partner). The possible range was 5 to 25 points, where a higher score indicates greater ambivalence in the relationship.

### 2.4. Data Analysis

The IBM SPSS Statistics program (version 25) was used to analyze the data, which obtained descriptive information, instrument reliability, an exploratory analysis of the ambivalence scale, and correlations between each of the variables. Then, to test the hypothetical research model (Figure 1), a structural equation model (SEM) was used in the STATA program (version 15.1), with a sequential multiple mediation model that simultaneously estimates multiple direct and indirect effects, with their standard errors (Full-information Maximum Likelihood) and Satorra-Bentler corrections; these corrections are used when the data is not normally distributed [47]. The model suggests that low sexual satisfaction and low emotional intimacy predict low satisfaction in the relationship, causing greater ambivalence which, in turn, could encourage the exercise of behaviors related to infidelity in social networks. To confirm if the model fit, the Chi Square Test was used (χ^2^_sb_), along with the Root Mean Square Error of Approximation (RMSEA_sb_), Standardized Root Mean Square Residual (SRMR), Tucker-Lewis Index (TLI_sb_), and Comparative Fit Index (CFI_sb_). RMSEA_sb_ values less than 0.08 and SRMR values less than 0.05 indicate an adequate adjustment of the model [48]. Likewise, CFI_sb_ and TLI_sb_ values greater than 0.95 represent a good fit of the model [48].

## 3. Results

### 3.1. Descriptive Analysis

The results reflected that 49% (*n* = 166) of the participants would be uncomfortable if their partner read the conversations they have with others on social networks, while 41% (*n* = 140) reported that if at some point their partners read the conversations they have with others on social networks, they could be annoyed. For their part, 53% (*n* = 180) indicated that they have had conversations on social networks that they prefer to hide from their partners and 35% (*n* = 120) prefer to hide things from their partners that they share with others on social networks. In turn, 28% (*n* = 97) prefer that their partners do not have access to their social networks because of some things they might find on them. In addition, the results showed that 27% (*n* = 93) prevent their partners from using their cell phone, for fear that they will access conversations they have had with others on social networks. Finally, 29% (*n* = 98) showed high levels of behaviors related to cyber infidelity [35].

### 3.2. Correlations

Spearman’s correlation coefficient analysis was performed for all measures, internal consistency indices were calculated, and means and standard deviations of the measures were obtained. The analyses revealed significant associations between infidelity-related behaviors in social networks and all the variables in the study (see Table 3).

### 3.3. Structural Model of Infidelity-Related Behaviors on Social Networks

**Figure 2:**
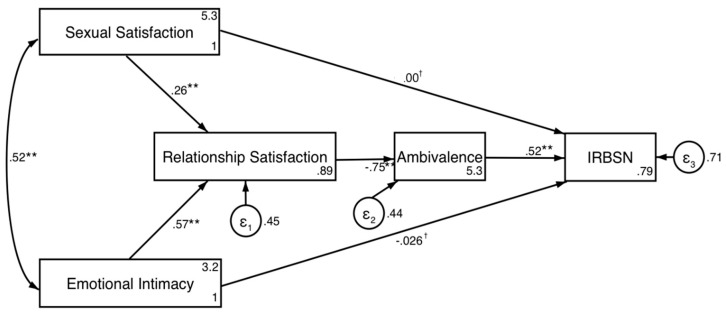
Model of infidelity-related behaviors on social networks. IRBSN = infidelity-related behaviors on social networks. Values with an asterisk (*) and cross (^†^) represent the standardized beta coefficients of regression. ** = *p* < 0.001; ^†^ = *p* > 0.05.

An SEM was then used to test the hypothesized research model of the impact of sexual satisfaction and emotional intimacy on the practice of infidelity-related behaviors on social media. Given that the data was not normally distributed (see Table 3), Satorra-Bentler adjustments were used to calculate the adjustment of the structural equation models, since the non-normality of the data changes the estimation errors and the global adjustment of the model [47]. According to the adjustment rates, the fit of our conceptual model is considered to be good (χ^2^_sb_ = 19.19 (3) *p* < 0.001, *RMSEA*_sb_ = 0.06 (CIs [0.04, 0.08]), *CFI*_sb_ = 0.99, *TLI*_sb_ = 0.97, *SRMR* = 0.03). Figure 2 shows the model and the respective standardized route estimates. According to the results, both sexual dissatisfaction (*β* = 0.26, 95% CIs [0.16, 0.35]) and low emotional intimacy (*β* = 0.57, 95% CIs [0.49, 0.65]) predict low satisfaction with the relationship. In turn, this low satisfaction causes greater ambivalence in the couple relationship (*β* = −0.75, 95% CIs [−0.80, −0.69]). In our model, this ambivalence in the relationship predicted a greater practice of infidelity-related behaviors on social networks (*β* = 0.52, 95% CIs [0.41, 0.63]). As for the direct effects of sexual satisfaction (*β* = 0.00, 95% CIs [−0.10, 0.10]) and emotional intimacy (*β* = −0.03, 95% CIs [−0.15, 0.09]), no significant effect was found for infidelity-related behaviors on social networks.

Bootstrapping analyses for indirect effects based on 1000 bootstrapping resamples and a 95% confidence interval were conducted for the indirect effects of sexual satisfaction and emotional intimacy on infidelity-related behaviors on social networks. As expected, significant indirect effects were observed between sexual satisfaction and infidelity-related behaviors on social networks (β = −0.12, 95% CIs [−0.17, −0.07]) and between emotional intimacy and infidelity-related behaviors on social networks (β = −0.37, 95% CIs [−0.48, −0.26]). These results confirm that satisfaction and ambivalence in the relationship completely mediate the relationship between sexual satisfaction and infidelity-related behaviors on social networks, as well as the relationship between emotional intimacy and infidelity-related behaviors on social networks.

## 4. Discussion

The purpose of this research was to present to the scientific community an explanatory model of how infidelity-related behaviors on social networks originated in a sample of Hispanic women. To this end, we developed a hypothesized model which proposes that low sexual satisfaction and low emotional intimacy will negatively affect satisfaction and stability in a relationship (ambivalence), significantly increasing the practice of the infidelity-related behaviors on social networks. In this model, we underline the mediating role of satisfaction and ambivalence in the relationship as a bridge of interaction between sexuality, emotional intimacy, and infidelity-related behaviors on social networks. Our results help us to theoretically and empirically understand how these dynamics develop in couple relationships, with the future objective of developing possible forms of prevention and intervention.

Surprisingly, 29% of participants showed high levels of ambiguous and misleading behaviors related to social network infidelity. This number significantly exceeds the 11% found in a sample of Americans [19]. These behaviors can be indicators of and precursors to explicit behaviors of cyber infidelity [4,34]. Specifically, these behaviors are: discomfort if the partner reads their conversations (49%), belief that the partner would be upset if they read their messages (41%), hiding conversations (53%), greater emotional intimacy with other people on social networks than with their partner (35%), and preferring that the partner does not have access to their social networks (29%) nor cell phone (27%). It is important to note that these behaviors are more common and acceptable than explicit behaviors [7], and have been shown to have pernicious and unfavorable effects on relationships and families [4].

Once the high prevalence of infidelity-related behaviors on social networks was confirmed in the sample, it was necessary to explain what factors promote or facilitate the exercise of them. Our linear structural equation model, which was validated by the results of this study, provides empirical evidence on the direct, pernicious, and unhealthy effect that a lack of sexual satisfaction and poor emotional intimacy can have on the quality of relationships in our sample. These results are consistent with what is stated by some theoretical models, such as the triangular theory of love [49], which indicates that two of the fundamental elements in stable relationships are emotional intimacy and passion (conceptualized in our study as sexual satisfaction). These elements are typical of two basic needs of the human being: affective need and sexual need. As a considerable amount of research [6,19,25,28], including our findings, have shown, when these needs are compromised, the result is dissatisfaction in the romantic relationship.

Consequently, our model suggests that, once the satisfaction in the relationship is lacerated, the individual will begin to experience emotions and feelings opposed to their partner, generating ambivalence in the relationship, as seen in our sample. That is, uncertainty about whether to maintain or leave the relationship. As our results demonstrated within our sample, these feelings of ambivalence play a significant role in the exercise of infidelity-related behaviors on social networks. This finding is consistent with the results of McDaniel et al. [4], who found that low satisfaction in the relationship and a high level of ambivalence were significantly related to infidelity behaviors through social networks. The novelty of our study was the inclusion of measures of sexual satisfaction and emotional intimacy. In this sense, our research expands the work done by McDaniel et al. [4], finding significant and inverse correlations between infidelity-related behaviors on social networks, sexual satisfaction, and emotional intimacy. Similarly, our study showed, like other studies [25,29], that emotional intimacy contributes more to a couple’s well-being than sexual satisfaction. In fact, some authors suggest that emotional distance when interacting with other variables such as unresolved conflicts determine a decrease in sexual satisfaction and an increase in infidelity [22]. Finally, our findings suggest that individuals with less sexual satisfaction, less emotional intimacy, and less relationship satisfaction are more likely to practice infidelity-related behaviors on social networks.

As for the theoretical implications of this study, it was shown that low sexual satisfaction and low emotional intimacy in our sample are risk factors that can undermine the quality of the romantic relationship, and indirectly increase the risk of practicing infidelity-related behaviors on social networks. Previous research has found that emotional connection and good communication are necessary for a good quality relationship [50]. In this sense, the negative effects of these variables could be minimized if the couple seeks to maintain emotional closeness and openness to dialogue on issues related to their sexuality. It has been established that communication within the couple is fundamental for its functioning and facilitates the expression of feelings, thoughts, fears, perception of the partner, negotiation, and problem-solving. Another important theoretical contribution of this study is that, for the first time in Latin America, it became apparent in our sample that satisfaction in the relationship and ambivalence completely mediate the relationship between sexual satisfaction, emotional intimacy, and infidelity-related behaviors on social networks.

As for the practical implications of the study, valuable information is provided for the design of interventions aimed at the prevention and reduction of infidelity-related behaviors on social networks. The findings propose several focuses in therapeutic practice with couples who are experiencing cyber infidelity: (1) thoroughly assess the sexual lifestyle of the partners and determine the levels of sexual satisfaction of the couple, and then intervene in this area, (2) develop strategies to improve effective communication, (3) strengthen emotional intimacy through therapeutic models focused on affective closeness (e.g., The Gottman Method of Relationship Therapy), and, (4) finally, institute behavioral norms on the use of social networks. It has been shown that when individuals identify that the other member of the relationship dialogues effectively, in a validating and close way, it increases willingness to engage good communication by fostering adequate levels of satisfaction in the relationship [51]. In preventive terms, it is vital that the couple, from the beginning, establish good bonds of communication and have openness towards dialogue around those issues related to sexuality and affectivity. Psychologists can use the findings of this study to psychoeducate couples during therapy, and need not wait for infidelity-related behaviors on social networks to come out in order to start discussing these issues.

It is worth noting that this study is the first in Latin America and the Caribbean to study the effects of sexual satisfaction and emotional intimacy on infidelity-related behaviors on social networks. However, some limitations were identified that readers and future researchers should consider. First, when a cross-sectional design has been used, readers should be wary of causal inferences, since it is unknown whether the results will be sustained over time. It is likely that the relationship between infidelity-related behaviors on social networks, sexual satisfaction, emotional intimacy, relationship satisfaction, and relationship ambivalence is bidirectional and reciprocal in our sample. This means that if we had built a model with the direction of causality completely reversed from their current theoretical model, we would have found an identical fit. For this reason, it is recommended to carry out longitudinal investigations that allow a more depth understanding of the phenomenon of infidelity-related behaviors on social networks. Second, our study was limited by respondents only being women. It may very well be that infidelity-related behaviors on social networks among men are affected in different ways by sexual satisfaction and emotional intimacy. This idea should be tested in future works. Third, the current study did not have dyadic data and so examining these same questions within dyadic samples is an extremely important future direction. Fourth, the type of sampling used does not allow generalization of the research results. Fifth, although online data collection has been shown to be reliable, valid, reasonably representative, cost-effective, and efficient [52], it may be limited with respect to the generalization of data given it generates an availability sample. Future research should consider conducting face to face interviews and using objective evidence of social media infidelities.

## 5. Conclusions

This research showed that there is a high prevalence (30%) of the infidelity-related behaviors on social networks among the Hispanic participants of this online study. Overall, women who practice infidelity-related behaviors on social networks showed less sexual satisfaction, less emotional intimacy, less relationship satisfaction, and greater ambivalence. The most important contribution of this research was the empirical validation of the explanatory model that we developed for this study. The model confirmed that low sexual satisfaction and low emotional intimacy negatively affect satisfaction and ambivalence, significantly increasing the practice of infidelity-related behaviors on social networks. Our results provide theoretical and empirical evidence on how infidelity-related behaviors on social networks develop in relationships, and could inform the development of possible forms of prevention and intervention.

## Figures and Tables

**Table 1:** Sociodemographic data of the sample.

	*n*	%
**Type of Relationship**		
Marriage	155	45.50%
Cohabiting (free union)	186	54.50%
**Academic Preparation**		
High school or less	24	7.00%
Associate degree/technical	86	25.20%
Bachelor’s degree	120	35.20%
Master’s degree	91	26.70%
Doctoral degree	20	5.90%
**Annual Income**		
$0–25,000	211	61.90%
$26,000–50,000	101	29.60%
$51,000–100,000	21	6.20%
$101,000 or more	8	2.30%

Note: *N =* 341.

**Table 2:** Items on relationship ambivalence, factorial loadings, and item discrimination indexes.

Items	*FL*	*r_bis_*
1. I feel ambivalent or insecure about continuing in my relationship with my partner.	0.89	0.77
2. I have considered ending my relationship with my partner or divorcing at some point during the last year.	0.95	0.80
3. I have talked with a close friend about the possibility of ending my relationship or obtaining a divorce.	0.84	0.75
4. If my relationship with my partner ends today, I can think of at least one person with whom I would like to start dating.	0.39	0.40
5. If my relationship with my partner ended today, I think I would not suffer much.	0.33	0.31

Note. *FL* = Factorial loadings of present study; *r_bis_* = item discrimination indexes.

**Table 3:** Correlations between variables, means, standard deviations, Shapiro–Wilk test, and Cronbach Alpha.

	*M*	*SD*	*SW*	*α*	*1*	*2*	*3*	*4*
1. Sexual satisfaction	56.78	10.82	0.99	0.89	-			
2. Emotional intimacy	23.84	7.44	0.98	0.90	0.50*	-		
3. Relationship satisfaction	24.85	6.05	0.97	0.92	0.56*	0.72*	-	
4. Relationship ambivalence	12.49	5.66	0.94	0.81	−0.41*	−0.66*	−0.75*	-
5. Infidelity-related behaviors	23.10	12.49	0.91	0.93	−0.22*	−0.35*	−0.42*	0.52*

Note. *M* = Mean; SD = standard deviation; α = Cronbach’s alpha coefficient; *SW* = Shapiro–Wilk; * = significant correlations *p* < 0.001. Degrees of freedom Shapiro–Wilk = 341, all values *p* < 0.001.
